# The Public Health in 1857
*From the Registrar-General's Quarterly Return.


**Published:** 1858-04

**Authors:** 


### SANITARY AND SOCIAL SCIENCE.

### THE PUBLIC HEALTH IN 1857.*

110,697 deaths were registered in the last quarter of the year 1857. This number exceeds by 14,176 the deaths in the corresponding quarter of 1856; and by 11,646 the average of the ten previous corresponding quarters. The mortality in the quarter was at the rate of 2·265 per cent. per annum, the average of the season being 2·167. The increase was equivalent to *one* on every 22 deaths. The increase of the mortality was greatest in the town districts or sub-districts, where 60,186 persons died, that is 6923 above the average 53,263; while the deaths in the country districts amounted to 50,511 or 4724 above the average, 45,787. After correcting for increase of population, on the assumption that the population in town and country increased at the same rates as in the ten years 1841-51, the mortality in the towns appears to be at the rate of 2·704 per cent., in the country at the rate of 1·926 per cent. per annum. The excess over the average of the season was ·182 in the towns, ·050 in the country; it was therefore more than three times as great in the towns as it was in the country districts.

The deaths in the year 1857 amounted to 420,019; and if the population of England and Wales is correctly estimated at 19,304,000 in the middle of that year, the rate of mortality was 2·176 per cent., or somewhat less than 22 to 1000 of the population. The average of the ten preceding years is 2·276 per cent.; consequently the mortality on the year 1857 was below the average.

The proportion of the deaths in a given time to a given population is not an exact measure of its vitality; the mortality being very different at different ages, and the proportional numbers of young and old being disturbed by excesses of births over deaths, and by emigration, the deaths in two equal populations may vary from differences in their composition as to age, without implying any real differences in the vitality. A disturbance may also be produced from disproportions in the sexes. Under ordinary circumstances the annual rate of mortality, however, at all ages, serves as a sufficiently accurate measure of the relative sanitary condition of the population; and where this is insufficient, the mortality at quinquennial or decennial periods of life may be separately determined.

* From the Registrar-General's Quarterly Return.

The mortality of England and Wales in 1857, has been compared with the mortality of England and Wales in the ten previous years, and it may be compared with the mortality (22·36 per 1000) in the 19 years 1838-56. It is below that average. But is that average itself, it may be asked, the true standard? What is the natural rate of mortality among Englishmen, under favourable sanitary conditions? Under such conditions how long do they live? How many of them die annually? No direct answer can be given to these questions. No large body of Englishmen is breathing pure air, living on a perfectly sound diet, free from all defilement, and free from vice, exercising duly the mind and the body generation after generation. We can point to no model city—to no model caste; we can discover no model parish in the country. In the matter of health we are all very ignorant, or desperately negligent. What courses then remain open to the inquirer? One only. The mortality of the districts of England in which the sanitary conditions are the least unfavourable, can be employed as the standard measure until happier times supply the real standard of vitality. Sixty-four districts, in various parts of the country, are found, where the mortality of the people ranged, on an average extending over ten years, from *fifteen* to *seventeen* deaths in 1000 living. This is not an accidental event; the mortality only fluctuates in such places slightly from year to year, and the death rate under the same circumstances will not be exceeded. The people dwell in sixty-four districts, extending over 4,797,315 acres, and their number at the last census was 973,070. Undoubtedly the sanitary conditions in which they live, are in many respects favourable. They generally follow agricultural pursuits; and they are scattered thinly over an open country, often on high ground, so that the impurities which they produce are dispersed and diluted in the air and water. They do not breathe each other's exhalations in theatres and churches. They do not drink water sullied by impurities. They do not drink poison in gin palaces. Their minds are not overwrought by dissipation, passion, intellectual effort. But visit their dwellings, and amidst much that is most commendable you will discover many sources of insalubrity. The bedrooms are often small, close, crowded; personal cleanliness is not much studied; the dirty pig, and filth of various kinds, lie here in close proximity to the house; the land there is imperfectly drained; in the winter, clothing, fuel, and food, are scantily enjoyed in all large or improvident families; ignorance yields its baneful fruits; medical advice is ill supplied or unskilful. Yet the annual mortality per 1000 of this million, men, women, and children, year after year, does not exceed 17. Is it not evident, that under more favourable auspices the death rate would be still lighter? Under such sanitary conditions as are known, and with all the mechanical appliances existing, can we not imagine a community living a healthier life than these isolated people?

Without affirming on physiological grounds that man was created to live a destined number of years, or to go through a series of changes, which are only completed in eighty, ninety, or a hundred years, experience furnishes us with a standard which can only be said to be too high. 17 in 1000 is supplied as a standard by experience. Here we stand upon the actual. Any deaths in a people exceeding 17 in 1000 annually are unnatural deaths. If the people were shot, drowned, burnt, poisoned by strychnine, their deaths would not be more unnatural than the deaths wrought clandestinely by disease in excess of the quota of natural death; that is, in excess of *seventeen* deaths in 1000 living.

But it may be said that this standard cannot fairly be applied to determine the excessive mortality of large towns, which can never become so healthy as the country. How healthy towns may become we do not know. It is only proved, that the population of parts of many towns experiences a mortality little above the natural standard; and that the prevalent diseases are referable to causes which, evidently from their nature, admit of removal. The question, however, is not, Does the excessive mortality admit of removal? but, does it exist?—and these two questions have no logical connection. The existence of the excess is established by comparing the actual mortality with the standard. Then the chief causes of the excessive mortality are now ascertained; and if the people have done all they can to remove them, the residual excess may be held to be inevitable. But what is inevitable at one time and in one place is not inevitable at other times and in other places. It is therefore of the utmost importance to keep steadily in view all the excessive mortality over and above that which is implied in the great decree: "It is appointed unto man once to die." In London, during the sixteenth century, the population lived about twenty years on an average, and 50 died out of 1000 living; consequently the excess over 17 was 33. That this excess was not inevitable is now demonstrated; for with a great increase in number, the population now lives about 37 years, and the mortality has fallen to 25 in 1000. Is the excess of 8 deaths a year among every 1000 living inevitable? This cannot be admitted for a moment, if we regard only the imperfect state of those sanitary arrangements which the public authorities of London have within their power. Nor can it be admitted that the excess of 5 deaths—or 22 deaths instead of 17—a year in every 1000 living, is inevitable in England and Wales, with evidence before our eyes of the same violations of the laws of nature in every district.

Whether the causes admit or do not admit of removal, the fact, then, is incontestable, and must not be lost sight of, that the excess of deaths in England and Wales over those from causes which exist in sixty-four districts, was 91,856 in the year 1857; for 420,019 persons died in that year, and about 328,163 persons would have died had the mortality not exceeded the standard of 17 deaths in 1000 living. Of the unnatural deaths, 18,328 happened in the country, or in the village districts, and 73,528 in the town districts. The portion of the loss of life falling during ten years in each of the 628 districts of the kingdom, has been published in an Annual Report, and deserves to be carefully studied.*

England is a great country, and has done great deeds. It has encountered in succession, and at times in combination, all the great powers of Europe; has founded vast colonies in America; and has conquered an empire in Asia. Yet greater victories have to be achieved at home. Within the shores of these islands the twenty-eight millions of people dwell who have not only supplied her armies, and set her fleets in motion, but have manufactured innumerable products, and are employed in the investigation of scientific truths, and the creation of works of inestimable value to the human race. These people do not live out half their days; *a hundred and forty thousand* of them die every year unnatural deaths; *two hundred and eighty thousand* are constantly suffering from actual diseases which do not prevail in healthy places; their strength is impaired in a thousand ways: their affections and intellects are disturbed, deranged, and diminished by the same agencies.† Who will deliver the nation from these terrible enemies? Who will confer on the inhabitants of the United Kingdom the blessings of health and long life? Who will give scope to the improvement of the English race, so that all its fine qualities may be developed to their full extent under favourable circumstances? His conquests would be wrought neither by wrong nor human slaughter; but by the application of the powers of nature to the improvement of mankind.

---

* Registrar-General's Sixteenth Annual Report, pp. 142-153.

† The annual number of deaths in the United Kingdom is about 616,000; and the number constantly sick is about twice the number of the annual deaths, or 1,232,000. If the annual rate of mortality per 1000 were reduced from 22 to 17, the deaths would fall to 476,000; the constantly sick to 952,000.

